# E-cigarette environmental and fire/life safety risks in schools reported by secondary school teachers

**DOI:** 10.1186/s12889-020-09319-8

**Published:** 2020-08-08

**Authors:** Maryanne L. Fakeh Campbell, Andrew Sansone, Lauren N. Gonzalez, Kevin R. J. Schroth, Derek G. Shendell

**Affiliations:** 1 New Jersey Safe Schools Program (NJSS), Rutgers School of Public Health (SPH), 683 Hoes Lane West, 3rd Floor SPH - Suite 399, Piscataway, New Jersey 08854 USA; 2 Department of Environmental and Occupational Health, Rutgers SPH, 683 Hoes Lane West, 3rd Floor SPH Building, Piscataway, New Jersey 08854 USA; 3 Department of Health Behavior, Society and Policy, Rutgers SPH, 683 Hoes Lane West, 3rd Floor SPH Building, Piscataway, New Jersey 08854 USA; 4 Center for Tobacco Studies, Rutgers SPH, New Brunswick, New Jersey 08903 USA; 5 Environmental and Occupational Health Science Institute (EOHSI), Rutgers Biomedical and Health Sciences, 170 Frelinghuysen Road, Piscataway, New Jersey 08854 USA

**Keywords:** Occupational health and safety, School health, Vulnerable sub-populations, Vaping, E-cigarettes

## Abstract

**Background:**

To identify if e-cigarette usage is an on-campus problem for secondary schools and evaluate initial school survey responses. More specifically, this survey can aid in identifying where students are seen using e-cigarettes, if smoke alarms have been newly inserted on school property, if smoke alarms have been tampered with to allow for vaping without detection; and, if any e-cigarette fires or explosions have occurred on school property.

**Methods:**

This survey, disseminated to New Jersey secondary school teachers across seven sessions January–July 2019, resulted in 104 complete responses of 109 respondents. The survey was conducted after fire prevention, exit/egress, and life safety portions of “OSHA 10 Plus for General Industry” courses. Survey questions included number of times teachers observed students vaping and location where vaping in last 12 months, fire alarm installation and tampering, and fires or explosions and injuries from vaping/e-cigarette devices.

**Results:**

Many (63%) respondents reported very or moderately common rates of vaping within their schools; however, three of four questions regarding school fire and smoke alarm use specifically to detect vaping had a majority of unsure/I don’t know responses.

**Conclusion:**

Results suggested concerns regarding student vaping and e-cigarette use inside and outside secondary schools. Improved school detection and response are warranted.

**Trial registration:**

Not applicable.

## Background

The New Jersey (NJ) Safe Schools Program (NJ SS) promotes classroom and workplace safety where NJ adolescents spend the most time by expanding knowledge and awareness of workplace safety and health issues by providing relevant resources and training to secondary schools within NJ.

The use of e-cigarettes, often also referred to as vaping, has continued to rise amongst adolescents [1–6]. The rates of current youth e-cigarette usage reported by National Youth Tobacco Survey among high school students increased from 1.5% in 2011 to 20.8% in 2018, and similarly use among middle school students increased from 0.6 to 4.9% [3]. In 2019, rates of youth e-cigarette usage increased to 27.5% among high school students and 10.5% among middle school students [7]. The growing trends in usage among youth have prompted the U.S. Centers for Disease Control and Prevention (CDC) to label e-cigarette use among young people as an emerging epidemic [6].

While it is illegal for people younger than age 21 to purchase e-cigarettes and vaping products, compliance and enforcement of minimum legal age laws are imperfect, e.g., at retail stores. The Food and Drug Administration (FDA) requires retailers to verify through photographic identification anyone younger than age 27, is at least 18 years old before they can purchase any tobacco purchases, including e-cigarettes [8]. Vendors, however, have not uniformly followed this requirement. For example, in one study where 18 and 19 year-olds were instructed to covertly attempt to purchase tobacco or e-cigarettes to observe identification requests, 49.8% of retail shops observed failed to verify the identification, and 44.7% of observed retail shops ultimately completed the purchase transaction without identification [9].

Attention has mostly been directed towards the potential long-term health effects of vaping; however, there is another environmental public health and occupational safety danger associated with the use of these products, i.e., fires and explosions. These events have the potential to be dangerous and safety and health risks could be exacerbated in school settings, where discrete e-cigarette product designs allow for unpermitted use likely done under concealment to avoid detection. Fire and explosion risk arise from devices with lithium batteries from an event known as thermal runaway. Thermal runaway can occur due to design flaws, low-quality materials, and improper use, leading to overheating of the battery [10–15].

As a result, there are sources of data emerging on injuries and property damage resulting from device fires and explosions [1, 4, 11–29]. For example, multiple reports have cited, collectively, several hundred incidences involving adolescents [4, 11]. One study included reported e-cigarette events from an online injury surveillance system from 2015 to 2017, which estimated a total of 2035 (1107-2964) incidents related to e-cigarette explosions and burn injuries presenting to emergency rooms [30]. Injuries resulting from device explosion or ensuing fire can range from minor injuries to even death. According to a 2019 U.S. Consumer Affairs report, for example, a 24-year-old male was killed by an exploding device after suffering from a severed artery caused by part of the device getting lodged in his throat. The man ultimately had a stroke and died as a result of the trauma [31].

Despite the increasing data on injuries caused by e-cigarette explosions, there is still a lack of media coverage and a knowledge gap associated with the dangers these devices present regarding fire and life safety. Warning labels concerning battery safety and the risk of fires and explosions on vaping products continue to be insufficient. As noted in one study, of the 24 top-selling devices sold, only ten contained a warning label about batteries, with only one of those being on the outside of the package [32]. The exclusion of a visible exterior warning label places more responsibility on the consumer to seek out this information, even if included in the package inserts or else online. Given the risks of fires and explosions are believed to come from the batteries within the devices, proper warnings should be prominently displayed to provide the necessary information needed for consumer protection; however, there is presently no requirement for battery warning labels.

Fire and explosion occurrences have prompted the FDA to release an infographic with five tips for preventing battery explosions: consideration of safety features, preventing metal objects from contacting batteries, utilizing the provided charger, monitoring charging, and replacing batteries as needed. Additionally, the FDA is now requesting submission of device failures through their generic online safety-based reporting tool for tracking purposes. The goal of reporting and tracking device failures is to help ensure the safety of these products both commonly and more increasingly used by Americans [33].

While long-term health effects of vaping are widely unknown, there is emerging evidence of acute health effects, such as “vape-associated pulmonary injury” (VAPI) and deaths linked to VAPI [34–37].

NJ SS conducted a survey, focusing on vaping and related environmental health, fire, and life safety concerns of e-cigarette use within secondary schools. The objectives of this initial study were to identify if e-cigarette usage is an on-campus problem within NJ secondary schools; to evaluate how schools are initially responding to this increasingly developing concern; and, to identify potential interventions for secondary schools.

## Methods

**Fig. 1:**
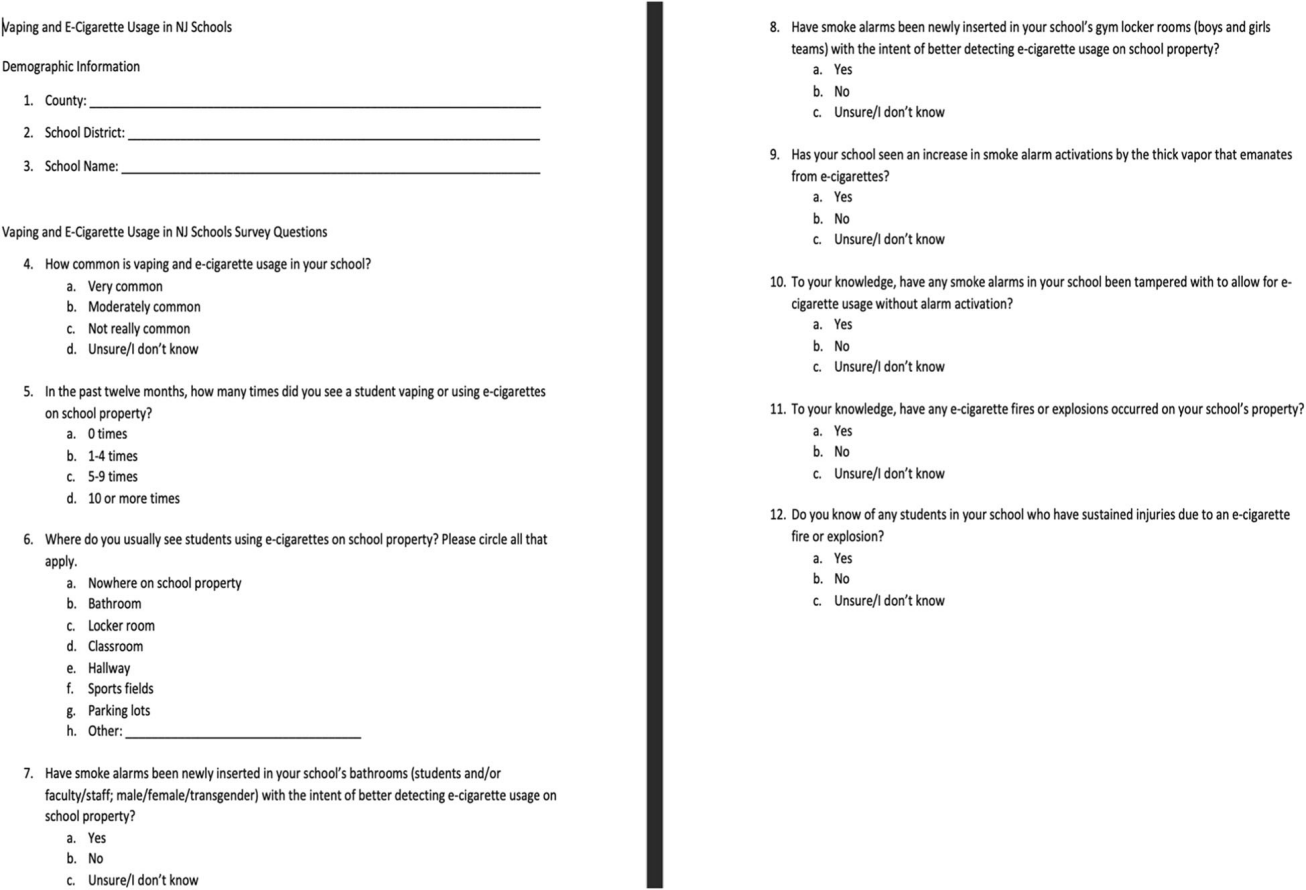
Survey content

The intent of the twelve question, multiple-choice survey put together by NJ SS is to identify if e-cigarette usage is an on-campus problem for NJ secondary schools, and evaluate how schools are initially responding, by state region. The survey was disseminated to NJ secondary school teachers immediately following the fire prevention, exit, and egress, and life safety module lectures of “OSHA (U.S. Department of Labor-Occupational Safety and Health Administration) 10 Plus for General Industry” course. The survey was completed during seven different training sessions of the “OSHA 10 Plus for General Industry” training courses that took place between January and July 2019, at multiple locations throughout NJ. Survey questions included county, school district, school name, prevalence, number of times teachers observed students vaping and location where vaping in last 12 months, fire alarm installation and tampering, and fires or explosions and injuries from vaping/e-cigarette devices (see Fig. 1).

Through the seven training sessions, there was a total of 109 participants, with 104 participants completing the entire survey. Four of the nonparticipants stated being a job coach hired from an outside entity, and the final nonparticipant stated working in an administrative setting without student observation as the reason for nonparticipation. Data collected were de-identified, but did ask for county, school district, and school name of current employment; overall, 103 survey participants provided this information.

Data were entered into PsychData and downloaded as a Microsoft Excel workbook. The initial analyses were performed within Excel and later confirmed and expanded upon with SAS version 9.4. Additionally, the counties were stratified into two regions (North and South). The “North” region included 11 of 21 NJ counties, with nine represented in this survey dataset: Bergen, Essex, Hudson, Hunterdon, Middlesex, Morris, Passaic, Union, and Somerset counties. The “South” region included the other ten NJ counties, with eight represented in this survey dataset: Atlantic, Burlington, Camden, Mercer, Cape May, Gloucester, Monmouth, and Ocean counties. Four counties within NJ did not have a participant in an “OSHA 10 Plus for General Industry” course in 2019; two from the North and two from the South: Warren and Sussex, and Cumberland and Salem, respectively.

## Results

The participants of this survey were highly concentrated in the “North” region of NJ, with a total of 75 out of 102 (74%). One participant did not provide a county, and an additional participant was excluded from stratification by region due to providing three different counties, with two being in the North (Bergen and Morris) and one is in the South (Mercer). A full breakdown based on participant county and State region is in Table 1.

**Table 1:** Participant’s region data

County	n
*South*	
Atlantic	2
Burlington	6
Camden	3
Cape May	1
Gloucester	5
Mercer	4
Monmouth	5
Ocean	2
*South Sub-total*^b^	27
*North*	
Bergen	19
Essex	8
Hudson	2
Hunterdon	1
Middlesex	9
Morris	8
Passaic	12
Somerset	10
Union	8
*North Sub-total*^b^	75
*Total*^a^	105

^a^There were 102/104 with one missing participant; however, above New Jersey counties total 105, which therefore includes the one participant who listed three New Jersey counties

^b^The two New Jersey Regions total 102, because this accounted for the one participant with three listed New Jersey counties and excluded one missing data

Differences by region were analyzed by Chi Square analysis and were generally not statistically significant (not included), which may be due to there being a statewide vaping issue not dependent on the region or to differences in respondent totals by region, among other factors not assessed. The breakdown of question responses by region were presented next to the total participant response in Tables 2 and 3 for comparison.

**Table 2:** Vaping prevalence and locations of use^a^

Question	Response	n	%	North New Jersey Region (n)	South New Jersey Region (n)
How common is vaping/e-cigarette usage in your school?	Very common	29	28	21	8
	Moderately common	36	35	23	12
	Not very common	23	22	19	3
	Unsure/I don’t know	16	15	12	4
In the past 12 months, how many times did you see someone vaping or using e-cigarettes on school property?	0 times	53	51	41	11
	1–4 times	39	37	25	13
	5–9 times	5	5	3	2
	10 or more times	7	7	6	1
Observed location of use					
*Bathroom*	No	47	45	39	7
	Yes	57	55	36	20
*Locker room*	No	92	88	67	23
	Yes	12	12	8	4
*Classroom*	No	95	91	69	24
	Yes	9	9	6	3
*Hallway*	No	93	89	67	24
	Yes	11	11	8	3
*Sports Field*	No	98	94	71	25
	Yes	6	6	4	2
*Parking lot*	No	73	70	56	15
	Yes	31	30	19	12
*Other*	No	96	92	69	25
	Yes	8	8	6	2

^a^There were 104 respondents to these questions

**Table 3:** School response and events

Question	Response	n	%	North New Jersey Region (n)	South New Jersey Region (n)
Have smoke alarms been newly inserted in your school’s bathrooms with the intent of better detecting e-cigarette usage on school property?	Yes	7	7	6	0
	No	38	36	25	13
	Unsure/ I don’t know	59	57	44	14
Have smoke alarms been newly inserted in your gym locker rooms with the intent of better detecting e-cigarette usage on school property?^a^	Yes	2	2	2	0
	No	30	30	19	11
	Unsure / I don’t know	68	68	51	16
Has your school seen an increase in smoke alarm activations by the thick vapor that emanates from e-cigarettes?^b^	Yes	2	2	2	0
	No	59	58	39	18
	Unsure / I don’t know	41	40	32	9
To your knowledge have any smoke alarms been tampered with to allow for e-cigarette usage without alarm activation?^b^	Yes	3	3	2	1
	No	40	39	28	10
	Unsure / I don’t know	59	58	43	16
To your knowledge have any e-cigarette fires or explosions occurred on your school’s property?^b^	Yes	0	0	0	0
	No	70	69	47	21
	Unsure / I don’t know	32	31	26	6
Do you know of any students in your school who have sustained injuries due to an e-cigarette fire or explosion?^b^	Yes	1	1	0	1
	No	65	64	45	18
	Unsure / I don’t know	36	35	28	8

^a^Overall, there were 100/104 participants who responded, with two missing, and two who explained in writing their schools do not have gym locker rooms. Also, at a region level there were 99 participants total because this accounted for the one participant with three listed New Jersey counties and excluded two missing, and two who explained in writing their schools do not have gym locker rooms

^b^Overall, there were 102/104 participants who responded, with two missing, and at region level there were 100 participants total because this accounted for the one participant with three listed New Jersey counties and excluded one missing data

The results of this survey suggested e-cigarette use is a common occurrence within NJ secondary schools (Table 2), with 65 of the 104 (63%) respondents reporting vaping being either very common or moderately common. However, detection of students vaping was low, with 53 out of 104 (51%) reporting observing a student vaping zero times in the last 12 months. This may be due to the abilities of students to conceal vaping devices through discrete product design as described in the introduction. Locations of observed usage ranged in frequency from a low six out of 104 (6%; sports field) to a high 57 out of 104 (57%; bathroom). However, the bathroom frequency creates a discrepancy with another question of the survey. When asked how many times they had observed a student vaping in the last 12 months on school property, 51 teachers reported observing a student one or more times while 57 reported observing a student vaping in a bathroom, resulting in a disagreement in answer choice by *n* = 6. Four of the six locations were indoor locations (bathroom, locker room, classroom, and hallway), while the other two were outdoor locations (sports field and parking lot). The most observed indoor location of student vaping was the bathroom, as stated above, and the most observed outdoor location was the parking lot with 31 out of 104 (30%). Additionally, “other” was provided as a response option with a line available for respondents to provide an additional location of where students were observed using e-cigarettes on-campus. Eight respondents (8%) selected “other,” and the locations listed by those selecting “other” included bus, cars, outside the front of the building, outside, stairwells, streets, and one wrote, “anywhere a teacher is not looking.”

Regarding initial school responses (Table 3), the survey asked questions about the installation of new smoke alarms for better detection within bathrooms and locker rooms, activation of alarms due to vapor, and whether alarms have been tampered with or not. These questions provided a higher return than expected of “unsure/I don’t know” responses, with that selection receiving a majority of the responses for three of the four questions. This includes in both whether alarms were newly installed in bathrooms (59/104, 57%) and locker rooms (68/100, 68%), as well as the question about fire alarm tampering to avoid detection (59/102, 58%). Two respondents did not reply to the locker room question due to their school not having locker rooms; this survey did not have a “does not apply” option. An additional two respondents did not reply and did not provide a reason. Two teachers reported an increase in smoke alarm activations because of vapor from e-cigarettes. While the majority reported “no” for an increase in activations (59/102, 58%), it is important to note how only seven and two respondents reported new alarms in bathrooms and locker rooms, respectively, for the purposes of better detecting e-cigarette use at their schools.

The final section of questions revolved around events of fires or explosions from vaping/e-cigarette devices and injuries presented in (Table 3). While rare, as stated earlier, it is also widely believed to be underreported. The media likely only reported extreme cases of exploding e-cigarettes resulting in actual severe injury and one fatality. Teachers responded to the question about knowing if fires or explosions have occurred on school property overwhelmingly with “no” (70/102, 69%), zero “yes,” responses, and the remainder being unsure. One respondent out of the 102 (1%) who responded to knowing if a student has been injured by a fire or explosion selected “yes,” while majority (65/102, 64%) responded “no.”

## Discussion

While there were not any known instances of fires or explosions by the respondents, there is always a threat for these events to take place. One teacher, however, noted how the school knew of a student who was injured by a device fire or explosion. The contents and size of the room where concealed use occur may result in more property damage or relatively more severe injuries. The indoor location with the greatest reported number of observed e-cigarette usage, the bathroom, also presents an added issue, considering the recent cases and deaths associated with VAPI [34, 35]. This may be an issue for not only the primary user, but also those exposed to second-hand vapor in tight corridors of the bathroom.

Additionally, the results of this study are observations by the teachers within their schools and may be underestimations of the true magnitude of the youth vaping crisis at secondary schools in NJ. The rates of vaping among youth have been steadily increasing; students interviewed at other schools in the U.S. reported vaping is everywhere and everyone does it; e-cigarettes are easy to conceal to avoid detection; and, one student was even recently quoted in reference to the 2019 National Youth Tobacco Survey results of one in four youths having tried vaping as being “low” [35]. Future research must explore the prevalence of vaping among youths, their perceived beliefs on individual risks, and health outcomes [36].

Each academic year in NJ schools runs for 9 months from September through June [38]. The results of the survey had seven respondents report observing a student vape on campus ten or more times in the past 12 months. Considering the school year only contains 9 months of in-session school, these seven respondents observed on average at least one student vaping each month. Schools throughout the U.S. are reporting taking extreme measures to reduce and even prevent vaping, including removing doors from bathroom stalls, forfeiting sport games, fines, and suspensions [35]. Additionally, the installation of new smoke alarms, including improved traditional smoke detectors wherever fire/life safety codes require them to be as well as potentially supplemental vaping aerosol detectors, may aid in the detection of vaping and e-cigarette usage in schools and in the overall reduction in vaping among students. While schools are starting to work to reduce the vaping problem, more efforts are needed, particularly on prevention, including through education and training about the known and potential unknown dangers of vaping.

There was a higher return of “unsure/I don’t know responses” than expected for questions about initial school responses for detection of vaping. This may be a reflection of the level of uncertainty among teachers pertaining to e-cigarette use and its relationship to fire and life safety. The relative lack of communication between safety and health professionals and the K-12 community on vaping to date may potentially be leading to a knowledge gap.

The survey had some notable strengths, including being easy to complete and required little time, about 5 min in person immediately after the relevant training topics of fire prevention, exit and egress, and life safety modules of the “OSHA 10 Plus for General Industry” course. Information obtained from the survey as used in this study provided a quick measure of the prevalence, frequency, and initial school response to vaping within secondary schools across the State of NJ. The data collected also provided areas for future focus for efforts on the detection and prevention of vaping within the secondary school setting.

The survey study also had known limitations. The “OSHA 10 Plus for General Industry” course is only required for teachers who are interested in obtaining their supervisor certification for structured learning (work-based learning) experiences, and thus limited this survey to only those participating in the training in 2019. Consequently, this cross-sectional survey did not employ a probability sample of NJ secondary schools and county career-technical-vocational school districts, although this purposeful convenience sample achieved statewide respondents, representative of the State of NJ and its 21 counties. Additionally, this study explicitly focused on vaping/e-cigarettes and did not directly assess overall use of various smoking products, including combustible cigarettes, and also did not directly assess student access to e-cigarettes. The survey was completed by teachers and the results reported their first-hand observances of student e-cigarette usage on school campuses. Finally, overall, 51 of the participants reported observing students vaping one or more times in the last 12 months, while 57 reported observing a student vaping in the bathroom in the last 12 months. These data suggested some disagreement among a few respondents.

## Conclusion

The results of this survey suggested a widespread vaping problem within NJ secondary schools, including indoor and outdoor locations. While there were limited respondents who reported known fires or explosions and injuries, there is great risk for future events to occur assuming the prevalence of vaping continues. There are therefore also environmental concerns for indoor air quality and secondhand exposure, considering the location with the greatest reported number of observed instances of e-cigarette use was within bathrooms. The results of this survey can provide a framework for better detection, prevention policies and procedures. For example, there can be interventions like the installation of new, improved traditional smoke detectors wherever school fire/life safety codes require them to be as well as additional vaping aerosol detectors--made available and affordable to K-12 schools--in more susceptible locations like bathrooms. Additionally, the results will be used in conjunction with the Rutgers School of Public Health Center for Tobacco Studies for the potential development of a future proposal for educational interventions and in-school research.

## Data Availability

This study’s data are secured on computers per IRB approved stewardship of NJ SS and/or are also publicly available from New Jersey Department of Education. Datasets used and analyzed during the current study are available from the corresponding author on reasonable request.

## Abbreviations

CDC: U.S. Centers for Disease Control and Prevention
NJ: State of New Jersey
NJ SS: New Jersey Safe Schools Program
OSHA: U.S. Department of Labor-Occupational Safety and Health Administration
VAPI: vape-associated pulmonary injury
